# Bioinformatic profiling identifies a platinum‐resistant–related risk signature for ovarian cancer

**DOI:** 10.1002/cam4.2692

**Published:** 2019-12-19

**Authors:** Ce Wu, Linxiu He, Qian Wei, Qian Li, Longyang Jiang, Lan Zhao, Chunyan Wang, Jianping Li, Minjie Wei

**Affiliations:** ^1^ Department of Pharmacology School of Pharmacy China Medical University Shenyang City China; ^2^ Liaoning Key Laboratory of Molecular Targeted Anti‐Tumor Drug Development and Evaluation China Medical University Shenyang City China; ^3^ Liaoning Cancer Hospital and Institute Cancer Hospital of China Medical University Shenyang City China; ^4^ Liaoning Blood Center Liaoning Provincial Key Laboratory for Blood Safety Research Shenyang China

**Keywords:** bioinformatics, high‐grade serous ovarian cancer, platinum resistance, prognosis

## Abstract

Most high‐grade serous ovarian cancer (HGSOC) patients develop resistance to platinum‐based chemotherapy and recur. Many biomarkers related to the survival and prognosis of drug‐resistant patients have been delved by mining databases; however, the prediction effect of single‐gene biomarker is not specific and sensitive enough. The present study aimed to develop a novel prognostic gene signature of platinum‐based resistance for patients with HGSOC. The gene expression profiles were obtained from Gene Expression Omnibus and The Cancer Genome Atlas database. A total of 269 differentially expressed genes (DEGs) associated with platinum resistance were identified (*P *< .05, fold change >1.5). Functional analysis revealed that these DEGs were mainly involved in apoptosis process, PI3K‐Akt pathway. Furthermore, we established a set of seven‐gene signature that was significantly associated with overall survival (OS) in the test series. Compared with the low‐risk score group, patients with a high‐risk score suffered poorer OS (*P* < .001). The area under the curve (AUC) was found to be 0.710, which means the risk score had a certain accuracy on predicting OS in HGSOC (AUC > 0.7). Surprisingly, the risk score was identified as an independent prognostic indicator for HGSOC (*P* < .001). Subgroup analyses suggested that the risk score had a greater prognostic value for patients with grade 3‐4, stage III‐IV, venous invasion and objective response. In conclusion, we developed a seven‐gene signature relating to platinum resistance, which can predict survival for HGSOC and provide novel insights into understanding of platinum resistance mechanisms and identification of HGSOC patients with poor prognosis.

## Graphical abstract

The present study aimed to develop a novel prognostic gene signature of platinum‐based resistance for patients with HGSOC. We developed a seven‐gene signature relating to platinum‐resistance, which can predict survival for HGSOC and provide novel insights into understanding of platinum‐resistance mechanisms and identification of HGSOC patients with poor prognosis.

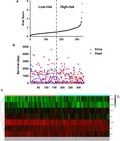

## 1 INTRODUCTION

Ovarian cancer is the most lethal gynecological malignancy in the world. It accounts for 2.5% of female malignant tumors, but 5% of cancer deaths due to low survival rates.^1^ There are four main histotypes of ovarian cancer: serous, endometrioid, mucinous, and clear cell.^2^ High‐grade serous ovarian cancer (HGSOC) has been thought to be the first leading histological subtype of ovarian cancer death. It is characterized by advanced stage at diagnosis and rapid progress.^3^ Combination chemotherapy with platinum compounds and taxanes for six cycles after cytoreductive surgery is a standard adjuvant treatment for patients with ovarian cancer. The majority of HGSOC patients responds well to platinum‐based chemotherapy. And 5‐year survival rates for ovarian cancer have largely improved from 40% to 47% over the last decades.^1^ Despite high validity of standard treatment, many patients suffer platinum resistance after initial response, which is one of the causes for low survival rate in HGSOC. Therefore, studying prognostic signatures will be pivotal to improve clinical treatment of HGSOC patients.

Currently, several biomarkers have been used to predict patients’ survival in HGSOC. For example, it has been well established that carbohydrate antigen 125 (CA125) is a dominant feature in diagnosis of ovarian cancer clinically.^4^ CA125 has provided useful information on identifying patients for secondary tumor‐debulking surgery, as well as treatment time for multiple conventional and novel drugs.^2^ Unfortunately, some studies have failed to define CA125 levels as an independent factor of prognosis or a target to improve overall survival (OS) of patients.^5^, ^6^, ^7^ Bioinformatics is fast becoming a key tool in helping investigators with new research ideas about cancer. Although there is a growing body of literature that recognizes the effect of mRNA expression signatures on recurrence^8^, ^9^ or OS,^8^, ^10^, ^11^, ^12^ there are few integrated analysis have studied the association between gene expression related to platinum resistance and OS of HGSOC.

In this study, the Gene Expression Omnibus (GEO) database^13^ was used to obtain differentially expression genes between platinum‐sensitive and resistant HGSOC tissue samples. The Cancer Genome Atlas (TCGA) database^14^ was used to identify a model consisted of mRNAs as a new indicator to predict outcome by analyzing the mRNA expression profiles and clinical features in HGSOC. Furthermore, the prognostic value of the indicator was also confirmed in patients with different clinical characteristics.

## 2 MATERIALS AND METHODS

### 2.1 Datasets and patients’ information

Four datasets (http://www.ncbi.nlm.nih.gov/geo/query/acc.cgi?acc=GSE51373,^15^ http://www.ncbi.nlm.nih.gov/geo/query/acc.cgi?acc=GSE32602,^16^ http://www.ncbi.nlm.nih.gov/geo/query/acc.cgi?acc=GSE65986,^17^ http://www.ncbi.nlm.nih.gov/geo/query/acc.cgi?acc=GSE26193 18) with tissue, histological type, histological grade, progression free survival (PFS), and drugs information were included for the analysis differentially expressed genes (DEGs) between platinum‐sensitive and resistant samples in HGSOC. Platform used in these datasets was GPL570 (Affymetrix Human Genome U133 Plus 2.0 Array). Platinum‐resistant disease was characterized by PFS of <6 months after the last platinum‐based treatment. Patients with PFS of >12 months had platinum‐sensitive disease.^2^ Overall, 104 HGSOC patients were selected for the analysis including data from 87 platinum‐sensitive samples and 17 platinum‐resistant samples (Table 1). The expression data and clinical data were downloaded from GEO Databases (https://www.ncbi.nlm.nih.gov/geo/). To test the prognostic significance of DEGs, gene expression data (level 3) of HGSOC patients from TCGA Databases (https://cancergenome.nih.gov/) with available clinical data were used.

**Table 1:** Top 10 up‐regulated differentially expressed genes (sorted by Fc)

mRNA	Official full name	Fc	*P*‐value
C2orf40	Chromosome 2 Open Reading Frame 40	1.62	.001
FAM84A	Family with Sequence Similarity 84, Member A	1.51	.003
NLGN4X	Neuroligin 4, X‐linked	1.32	.004
CMBL	Carboxymethylenebutenolidase Homolog (Pseudomonas)	1.19	.001
CNTN3	Contactin 3 (plasmacytoma associated)	1.18	.006
MUM1L1	Melanoma Associated Antigen (mutated) 1‐like 1	1.18	.012
PCP4	Purkinje Cell Protein 4	1.17	.020
SOX11	SRY (sex determining region Y)‐box 11	1.10	.033
TCEAL2	Transcription Elongation Factor A (SII)‐like 2	1.09	.019
PCSK1N	Proprotein Convertase Subtilisin/kexin Type 1 Inhibitor	1.07	<.001

### 2.2 Identification of DEGs in HGSOC samples

Linear models for microarray data (limma) package^19^ were used for differential expression analysis. Robust multichip average (RMA) method was used for data normalization. Imputation for microarray data (impute) package^20^ was used to impute missing expression data. Surrogate variable analysis package^21^ was used for removing batch effects and other unwanted variations in experiment. Expression difference was characterized by fold change (Fc) and *P*‐valve. Fc was defined by the ratio between platinum‐resistant group and sensitive group in expression value of each gene. Genes with Fc＞1.5 and *P* < .05 were defined as DEGs.

### 2.3 Gene annotation and pathway enrichment analyses

The Database for Annotation, Visualization and Integrated Discovery (DAVID, https://david.ncifcrf.gov/home.jsp)^22^ was used to perform gene function annotation and pathway enrichment analysis. Gene annotations were performed from three aspects, namely, cellular component, biological process, and molecular function by gene ontology. KEGG (Kyoto Encyclopedia of Genes and Genomes)^23^ pathway enrichment analyses were also performed to investigate the underlying functions of aforementioned DEGs. *P* < .05 was used as the significance level.

### 2.4 Construction of the DEGs‐based prognostic model and data analysis

Package survival^24^was used for Cox proportional hazard regression to construct the prognostic model. The univariate regression with *P *< .05 was used to identify OS related mRNAs from the DEGs. The multivariate regression was used to confirm mRNAs selected by that univariate regression could be brought into prognostic model and calculate risk score. The risk score of each patient was calculated according to the formula: risk score = *h*0 (*t*) exp (*β*1*X1 + *β*1*X1 + … βn*Xn) (*X*: gene expression value; *β*: the coefficient derived from multivariate regression; *h* (0): baseline hazard function, not specified). HGSOC patients were divided into high‐risk group and low‐risk group according to the median risk score.^25^ Survival curves were created using survfit functions. And survdiff function was used to test survival curve differences. Package survival receiver operating characteristic (ROC)^26^ was used to create ROC curve to assess the predictive accuracy of the risk score for time‐dependent disease outcomes within 3 years. Cox regression was also performed to evaluate the effect and independence of risk score and clinicopathological parameters, including age, histological grade, stage, residual tumor size, anatomic neoplasm subdivision, and treatment outcome on OS of HGSOC patients.

## 3 RESULTS

### 3.1 Identification of DEGs involved in platinum‐resistant HGSOC and pathway enrichment analyses

**Figure 1:**
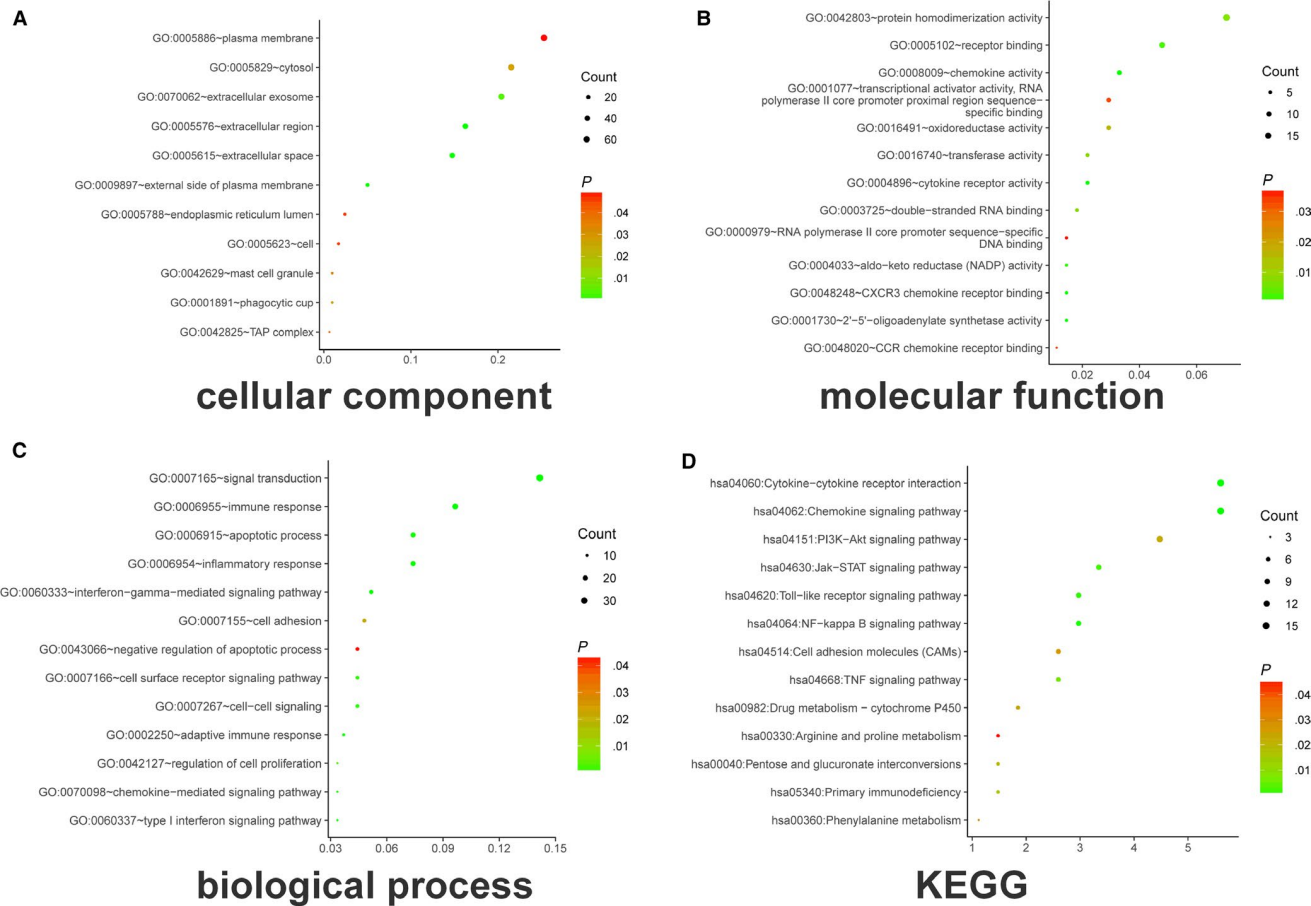
Differentially expressed genes were enriched in (A) cellular component, (B) molecular function, (C) biological process, and (D) Kyoto Encyclopedia of Genes and Genomes (KEGG) pathway (more details are presented in Table S1)

The gene expression profile with accession numbers http://www.ncbi.nlm.nih.gov/geo/query/acc.cgi?acc=GSE51373, http://www.ncbi.nlm.nih.gov/geo/query/acc.cgi?acc=GSE32062, http://www.ncbi.nlm.nih.gov/geo/query/acc.cgi?acc=GSE65986, and http://www.ncbi.nlm.nih.gov/geo/query/acc.cgi?acc=GSE26193 were downloaded from GEO database. The volcano plot (Figure S1A) showed the difference of mRNA expression between platinum‐sensitive (n = 87) and resistant group (n = 17). Compared with the sensitive group, 269 DEGs were obtained including 123 up‐regulated and 146 down‐regulated in the resistant group (*P* < .05, Fc > 1.5). The heatmap presented the expression of DEGs with *P* < .05 and Fc > 1.5 (Figure S1B). The top 10 up‐regulated and down‐regulated DEGs (sorted by Fc) are displayed in Tables 1 and 2. Then we performed gene function analysis through the web‐tool: DAVID. As shown in Figure 1, DEGs were significantly enriched in cellular components (Figure 1A), protein homodimerization activity (Figure 1B), signal transduction, immune response, and apoptotic process (Figure 1C). And KEGG analysis (Figure 1D) showed that DEGs were enriched in the PI3K‐Akt signaling pathway, drug metabolism‐cytochrome P450, etc, *P* < .05 was used as the significance level.

**Table 2:** Top 10 down‐regulated differentially expressed genes (sorted by Fc)

mRNA	Official full name	Fc		*P*‐value
IGLC1	Immunoglobulin Lambda Constant 1 (Mcg marker)		2.21	.003
CXCL10	Chemokine (C‐X‐C motif) Ligand 10		1.51	.003
CXCL11	Chemokine (C‐X‐C motif) Ligand 11		1.49	.007
HLA‐DRB4	Major Histocompatibility Complex, Class II, DR beta 4		1.25	.038
CXCL9	Chemokine (C‐X‐C motif) Ligand 9		1.23	.016
CXCL13	Chemokine (C‐X‐C motif) Ligand 13		1.17	.010
CXCL8	Chemokine (C‐X‐C motif) Ligand 8		1.17	.008
GBP1	Guanylate Binding Protein 1, Interferon‐inducible		1.16	<.001
ADAMDEC1	ADAM‐like, Decysin 1		1.14	.012
SAMD9	Sterile Alpha Motif Domain Containing 9		1.11	.001

### 3.2 Construction of DEG‐based prognostic model

To explore whether DEGs are related to OS, 333 HGSOC samples with the expression profile in TCGA databases were selected for the Cox regression analysis. Univariate Cox regression showed that 12 of 269 DEGs were determined to be associated with OS in HGSOC significantly (*P* < .05, Table 3), while multivariate Cox regression indicated that seven DEGs were included in the prognostic model (Table 3). Among them, LYPD6B, CD38, CMBL, and KIAA2022 were independent protective factors due to hazard ratio (HR) being less than 1. On the contrary, LYRRC17, ZHX3, and AKR1B10 were independent risky factors for OS in HGSOC patients, as the HR were greater than 1.

**Table 3:** Univariate and Multivariate analysis associated with overall survival in patients with high‐grade serous ovarian cancer

mRNA	Univariate Cox regression			Multivariate Cox regression		
	*β* (Cox)	*P*	HR (95% CI)	*β* (Cox)	*P*	HR (95% CI)
CD38	−0.122	.007	0.885 (0.811‐0.967)	−0.143	.002	0.866 (0.793‐0.947)
FCGBP	0.110	.013	1.117 (1.023‐1.219)			
PDK4	0.184	.017	1.202 (1.033‐1.398)			
CXCL13	−0.068	.022	0.934 (0.881‐0.990)			
ATP1A2	0.079	.024	1.082 (1.010‐1.159)			
AKR1B10	0.074	.032	1.077 (1.006‐1.153)	0.101	.006	1.106 (1.030‐1.187)
LYPD6B	−0.116	.033	0.890 (0.800‐0.990)	−0.121	.031	0.886 (0.794‐0.989)
ADAMDEC1	−0.065	.033	0.937 (0.882‐0.995)			
LRRC17	0.108	.037	1.115 (1.007‐1.234)	0.170	.005	1.185 (1.052‐1.336)
CMBL	−0.104	.041	0.901 (0.815‐0.996)	−0.159	.003	0.853 (0.768‐0.948)
ZHX3	0.206	.043	1.229 (1.007‐1.499)	0.235	.041	1.265 (1.010‐1.584)
KIAA2022	−0.076	.045	0.927 (0.861‐0.998)	−0.124	.004	0.883 (0.811‐0.962)

### 3.3 The risk score as an independent prognosis indicator in HGSOC

**Figure 2:**
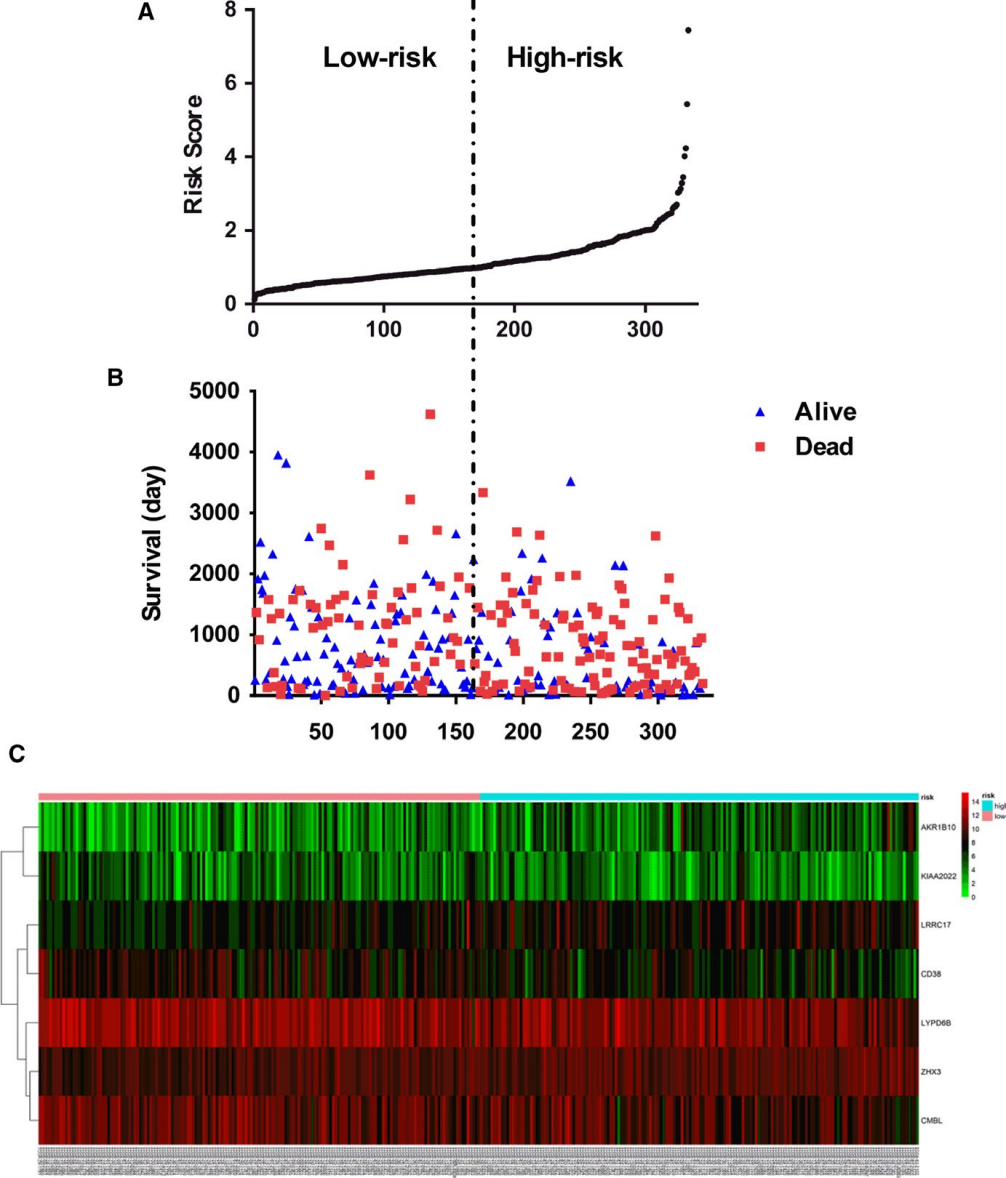
Seven differentially expressed gene (DEG) signatures related to risk score predict overall survival in the patients. A, DEG risk score distribution in each patient. B, Survival days of patients in order of the value of risk scores. C, A heatmap of seven selected genes’ expression profile

**Figure 3:**
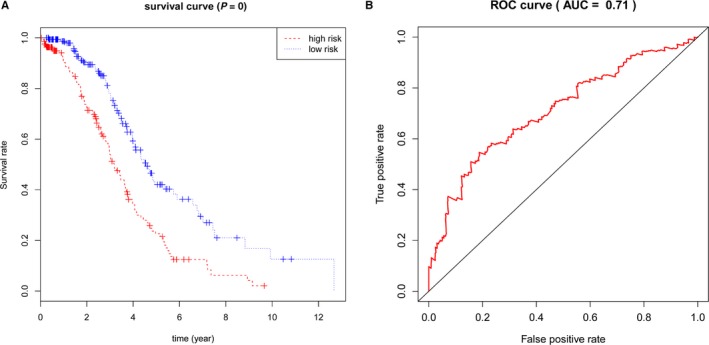
Kaplan‐Meier survival analysis for the patients with high‐grade serous ovarian cancer (HGSOC) in The Cancer Genome Atlas (TCGA) dataset. A, The Kaplan‐Meier curve for patients with HGSOC divided into high‐risk and low‐risk. B, Receiver operating characteristic curve in discriminating patients with high‐risk score from those with low‐risk score (AUC = 0.71)

Risk scores for each patient were calculated according to the formula mentioned in the materials and methods. All patients were divided into two groups: low‐risk group (n = 167) and high‐risk group (n = 166) by the median value of risk score (Figure 2A). Patients survival status is shown in Figure 2B. The different expression patterns of the seven DEGs in the low‐ and high‐risk groups are showed in heatmap (Figure 2C). As the value of risk score increased, the expression of protection factors tend to decrease, while the risky mRNAs tend to increase in expression. The survival curves revealed patients with high‐risk scores suffered poorer prognosis compared with the low‐risk group (*P* < .001, Figure 3A). The most striking result to emerge from the data is that the area under ROC curve (AUC) was 0.710 (Figure 3B), indicating that the risk score had a certain accuracy on predicting 3‐year OS in HGSOC. In addition, to determine whether the prognostic function of the risk score derives from one mRNA, we drew survival curves (Figure S2) and ROC curves (Figure S3) of every single mRNA. It is regrettable that none of these mRNAs was an accurate predictor of OS in HGSOC with AUC for ROC <0.7, which means the prognostic value of risk score was higher than any single mRNA.

Moreover the prognostic role of the novel risk score was also evaluated together with the classical clinical pathological parameters that might influence the prognosis of HGSOC. Univariate and multivariate Cox regression analysis indicated that only no‐meeting‐objective response (HR = 2.623, *P* < .001) and high‐risk score (HR = 1.899, *P* < .001) were independent prognostic indicators for OS (Table 4).

**Table 4:** Univariate and multivariate analysis for each clinical feature

Pathological parameters	Univariate Cox regression			Multivariate Cox regression		
	*β* (Cox)	*P*	HR (95% CI)	*β* (Cox)	*P*	HR (95% CI)
Age (>58/≤58) 163/170	0.037	.809	1.038 (0.768‐1.403)			
Grade (G3 ~ 4/G2) 294/39	0.351	.127	1.421 (0.905‐2.229)			
Histological stage (III ~ IV/II) 311/21	0.848	.062	2.336 (0.958‐5.695)			
Residual tumor (>10/≤10 mm) 84/219	0.225	.185	1.253 (0.898‐1.749)			
Anatomic neoplasm subdivision (bilateral/unilateral) 230/87	0.320	.081	1.377 (0.962‐1.972)			
Objective response (no/yes) 46/197	0.970	<.001	2.639 (1.744‐3.994)	0.964	<.001	2.623 (1.732‐3.971)
Venous invasion (yes/no) 58/38	−0.244	.471	0.784 (0.404‐1.520)			
Lymphatic invasion (yes/no) 91/45	0.350	.220	1.419 (0.811‐2.482)			
Risk score (high/low) 166/167	0.733	<.001	2.082 (1.530‐2.834)	0.641	<.001	1.899 (1.359‐2.653)

### 3.4 Prognosis effect assessment of risk score in subsets of HGSOC patients

**Figure 4:**
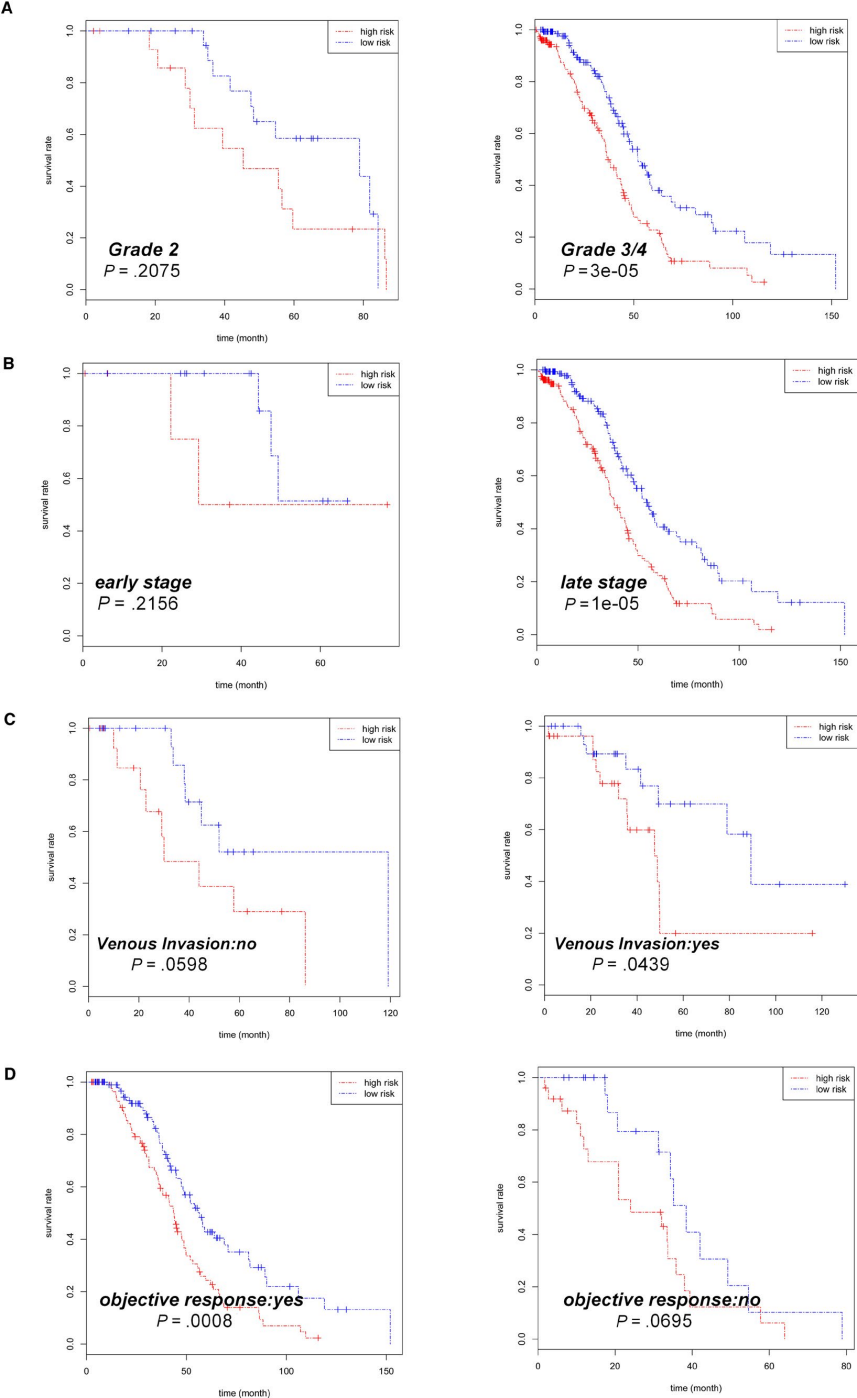
Kaplan‐Meier curves for prognostic value of risk‐score signature for patients divided by each clinical feature. A, Grade. B, Stage. C, Venous invasion. D, Objective response

**Figure 5:**
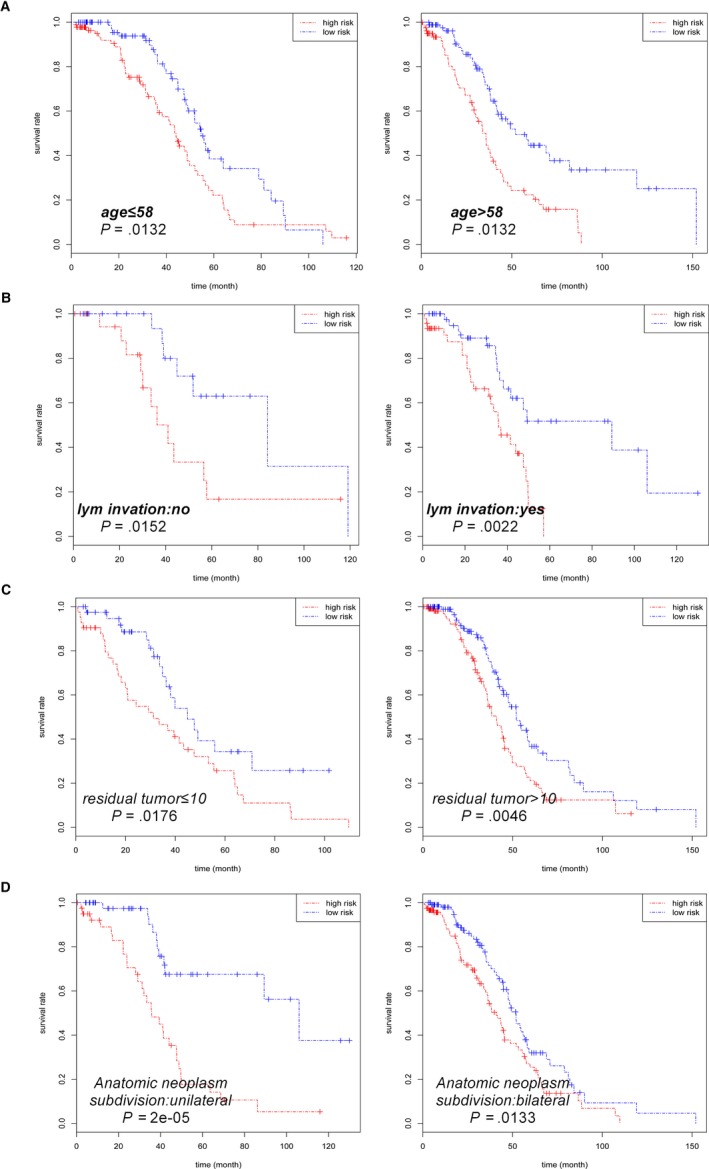
Kaplan‐Meier curves for prognostic value of risk‐score signature for patients divided by each clinical feature. A, Age. B, Lymphatic invasion. C, Residual tumor size. D, Anatomic neoplasm subdivision

Last but not the least, the prognostic value of the risk score in the OS of HGSOC patients was also evaluated in subsets of HGSOC patients with different clinical characteristics, and the results obtained from the analysis are shown in Table 5. We analyzed the correlation between risk score and OS in different subgroups, such as age (≤58 or >58), histologic grade (high or low differentiation), clinical stage (early or advanced stage), lymphatic invasion, venous invasion, treatment outcome objective response ([OR] or no‐meeting‐objective response), residual tumor size, and anatomic neoplasm subdivision (unilateral or bilateral). Subgroup analyses demonstrated that high‐risk score was associated with poor prognostic in the patients with grade 3‐4 (*P* < .001, Figure 4A), advanced stage (*P* < .001, Figure 4B), venous invasion (*P* = .0439, Figure 4C), and OR subsets (*P* < .001, Figure 4D) closely. In addition, there were strong relationships between the high‐risk score and low survival rate in different age groups (Figure 5A), lymphatic invasion (Figure 5B), residual tumor size (Figure 5C), anatomic neoplasm subdivision (Figure 5D). But no significant differences were found between the subgroups in these features.

**Table 5:** Correlation of risk scores with overall survival (OS) in subsets of different clinical features

3‐y OS rate % (95% CI)	High risk	Low risk	*P‐*value	Number (high/low)
Age ≤58	63.0 (52.0‐76.1)	85.5 (76.5‐95.5)	.013	87/83
Age >58	44.6 (33.6‐59.2)	71.8 (61.4‐83.9)	<.001	79/84
Grade 2	62.3 (40.9‐94.9)	88.5 (74.8‐100.0)	.208	16/23
Grade 3‐4	52.9 (44.3‐63.2)	76.1 (68.1‐85.1)	<.001	150/144
Stage II	50.0 (18.8‐100.0)	—	.216	6/15
Stage III ~ IV	54.6 (46.2‐64.4)	75.9 (68.1‐84.5)	<.001	159/152
Residual tumor ≤10 mm	57.6 (47.6‐69.7)	78.2 (69.2‐88.4)	.005	113/106
Residual tumor >10 mm	46.6 (33.0‐65.8)	68.3 (52.6‐88.6)	.018	42/42
Anatomic neoplasm subdivision (unilateral)	48.2 (33.7‐69.0)	90.2 (80.1‐100.0)	<.001	41/46
Anatomic neoplasm subdivision (Bilateral)	58.2 (48.7‐69.5)	73.1 (63.8‐83.9)	.013	119/111
Objective response: yes	62.1 (52.3‐73.9)	80.8 (72.5‐90.1)	.001	93/104
Objective response: no	24.6 (10.9‐55.5)	51.1 (28.7‐90.8)	.070	25/21
Lymphatic invasion: no	58.4 (37.4‐91.3)	93.3 (81.5‐100.0)	.015	22/23
Lymphatic invasion: yes	49.0 (34.3‐70.0)	74.0 (59.7‐91.8)	.002	46/45
Venous invasion: no	48.4 (26.3‐88.8)	85.7 (69.2‐100.0)	.060	17/21
Venous invasion: yes	59.9 (41.3‐86.8)	83.3 (69.2‐100.0)	.044	26/32

## 4 DISCUSSION

High‐grade serous ovarian cancer is the main histological type that causes death in patients with ovarian cancer. Therefore, studying signatures with prognostic value would be critical for treatment improvement of HGSOC patients. In this study, a potential platinum resistant‐related risk signature was built and evaluated to predict survival in HGSOC according to the results of DEGs identification involved in platinum‐resistant and Cox proportional regression analysis. The risk score computed according to coefficients and expressions of seven mRNAs, including LRRC17, ZHX3, CD38, AKR1B10, LYPD6B, KIAA2022, and CMBL, could serve as an accurate and independent prognostic indicator for HGSOC. Several single genes were shown to be a powerful in assessment of prognosis for HGSOC. For example, some studies focused their point on the prognostic value of apolipoprotein B mRNA editing enzyme catalytic subunit 3 (APOBEC3) and folate receptor 1 (FOLR1).^27^, ^28^ Similarly, a study highlighted the prognostic value of a 11‐gene model which was obtained by Rebust likelihood‐based study on ovarian samples from TCGA.^29^ Despite those surprising findings, no biomarkers for prognosis prediction of HGSOC were yet in clinical use. However, there was no large scale genomic analysis studied the collaborative effect of multiple genes related to platinum resistance on the prognosis in HGSOC. Therefore, it is extremely important to find and design a new model that can predict the prognosis of platinum resistance in HGSOC and with which we can predict the prognosis of patients with HGSOC and take corresponding treatment measures.

Platinum‐based chemotherapy combined with debulking surgery is the standard therapy for ovarian cancer patients. Despite high response rate to initial therapy, many patients would suffer platinum resistance and relapse after that, which lead to poor prognosis. So, we attempt to construct a potential platinum‐resistant focused model to predict the OS in HGSOC patients. Firstly, a total of 269 DEGs between platinum‐resistant and sensitive HGSOC tissue samples were obtained. To understand the functions of DEGs, gene function analysis was performed. DEGs were involved in many biological processes related to drug resistance, such as signal transduction and apoptosis process. They may also participate in the drug resistance of ovarian cancer through PI3K‐Akt signaling pathway and drug metabolism—cytochrome P450. Next, a prognosis model consisted of seven DEGs (LRRC17, ZHX3, CD38, AKR1B10, LYPD6B, KIAA2022, and CMBL) was constructed based on relationships between DEGs and the OS in 333 HGSOC patients from TCGA. Among them, LYPD6B, CD38, CMBL, and KIAA2022 were regarded as independent protective factors, while others were defined as risky factors. Surprisingly, survival and ROC curve analyses revealed that the risk score generated from the prognosis model could serve as an indicator of the OS in HGSOC with certain accuracy. Moreover the independence of prognosis effect was also confirmed through comparing the novel risk score with classical clinical pathological parameters.

Among the identified seven genes (LRRC17, ZHX3, CD38, AKR1B10, LYPD6B, KIAA2022, and CMBL) in our study, CD38 was well known about its correlation with hematological malignancies. High expression of CD38 was associated with shorter lymphocyte doubling time and poor prognosis in chronic lymphocytic leukemia^30^ In addition, several studied argued that CD38 played vital role in multiple myeloma.^31^, ^32^, ^33^ AKR1B10 is a member of aldo‐keto reductase family 1 member B subfamily and expressed in normal epithelial cells of the digestive tract as a cytosolic reductase.^34^ It has been observed that AKR1B10 was associated with survival in gastric cancer, colorectal cancer, and hepatocellular carcinoma.^35^, ^36^, ^37^ To date, Very little is currently known about AKR1B10 in ovarian cancer. Hong et al found that the low expression of LRRC17 was a risk factor for fracture in postmenopausal women.^38^ However, there is little published data on LRRC17 function in cancer.^39^, ^40^ Ochoa et al^41^, ^42^ suggested that LYPD6B enhanced the sensitivity of (α3)3(β4)2 nicotinic acetylcholine (ACh) receptors to ACh. CMBL, which is a highly expressed in liver cytosol, was the primary cysteine hydrolase to bioactivate Olmesartan Medoxomil in the liver and intestine.^43^, ^44^ Existing research has recognized the critical role of KIAA2022 in X‐linked mental retardation (XLMR) and brain development.^45^, ^46^, ^47^, ^48^, ^49^ KIAA2022 enhanced cell adhesion and migration by regulating adhesion molecules expression, such as N‐Cadherin and β1‐integrin.^47^, ^50^ ZHX3 is a member of the zinc fingers and homeoboxes (ZHX) gene family. Although it is now well established that ZHX1 and ZHX2 worked as tumor suppressors,^51^, ^52^, ^53^ the role of ZHX3 in cancer is not clear. Studies showed that ZHX3 was upregulated and might be an independent indicator of the OS in renal clear cell carcinoma.^54^, ^55^

In the final part of this study, the prognosis efficacy of risk score was also evaluated in subsets of HGSOC patients with different clinical characteristics. Subgroup analyses demonstrated that high‐risk score was associated with shorter OS closely in the Grade 3‐4, late stage, venous invasion, and OR subsets. There also were strong relationships between the high‐risk score and low survival rate no matter what the age, lymphatic invasion, residual tumor size, anatomic neoplasm subdivision are. These findings, while preliminary, have important implications for developing platinum‐resistant focused indicator for survival assessment in HGSOC. The risk score will provide a new direction for the evaluation of HGSOC prognosis, which is different from the traditional assessment system. It might be helpful for individualized treatment and survival improvement through further patient stratification.

Additionally, some limitations of this study should be considered. First, the sample size in this study is small, so further validations with larger samples and other experimental methods are quite essential. Second, this study was limited to the analysis of mRNA expression profile without considering interactions with miRNA, lncRNA, and other factors, further comprehensive study are expected to be done. Third, previous studies of LRRC17, ZHX3, LYPD6B, KIAA2022, and CMBL in cancer still remain paucity despite the importance of them. Extensive researches are required to answer this question.

In conclusion, to the best of our knowledge, we have identified seven genes associated with platinum resistance in HGSOC patients using the Cox regression model. Further analysis revealed that the seven‐gene signature could be an independent factor predicting the prognosis of the platinum resistance in HGSOC patients. These findings may be a potential biomarker for the prognosis of platinum resistance and provide insights into theoretical guidance and decision making in clinical practice of HGSOC.

## CONFLICT OF INTEREST

None declared.

## References

[1] Torre LA , Trabert B , DeSantis CE , et al. Ovarian cancer statistics, 2018. CA Cancer J Clin. 2018;68(4):284‐296. 29809280 10.3322/caac.21456 PMC6621554

[2] Romero I , Bast RC Jr . Minireview: human ovarian cancer: biology, current management, and paths to personalizing therapy. Endocrinology. 2012;153:1593‐1602. 22416079 10.1210/en.2011-2123 PMC3320264

[3] McPherson A , Roth A , Laks E , et al. Divergent modes of clonal spread and intraperitoneal mixing in high‐grade serous ovarian cancer. Nat Genet. 2016;48:758‐767. 27182968 10.1038/ng.3573

[4] Yin BW , Lloyd KO . Molecular cloning of the CA125 ovarian cancer antigen: identification as a new mucin, MUC16. J Biol Chem. 2001;276:27371‐27375. 11369781 10.1074/jbc.M103554200

[5] Makar AP , Kristensen GB , Kaern J , Bormer OP , Abeler VM , Trope CG . Prognostic value of pre‐ and postoperative serum CA 125 levels in ovarian cancer: new aspects and multivariate analysis. Obstet Gynecol. 1992;79:1002‐1010. 1579296

[6] Scholl SM , Bascou CH , Mosseri V , et al. Circulating levels of colony‐stimulating factor 1 as a prognostic indicator in 82 patients with epithelial ovarian cancer. Br J Cancer. 1994;69:342‐346. 8297732 10.1038/bjc.1994.62 PMC1968706

[7] Venesmaa P , Lehtovirta P , Stenman UH , Leminen A , Forss M , Ylikorkala O . Tumour‐associated trypsin inhibitor (TATI): comparison with CA125 as a preoperative prognostic indicator in advanced ovarian cancer. Br J Cancer. 1994;70:1188‐1190. 7981075 10.1038/bjc.1994.471 PMC2033663

[8] Mankoo PK , Shen R , Schultz N , Levine DA , Sander C . Time to recurrence and survival in serous ovarian tumors predicted from integrated genomic profiles. PLoS ONE. 2011;6:e24709. 22073136 10.1371/journal.pone.0024709 PMC3207809

[9] Miller KR , Patel JN , Zhang Q , et al. HOXA4/HOXB3 gene expression signature as a biomarker of recurrence in patients with high‐grade serous ovarian cancer following primary cytoreductive surgery and first‐line adjuvant chemotherapy. Gynecol Oncol. 2018;149:155‐162. 29402501 10.1016/j.ygyno.2018.01.022

[10] Bonome T , Levine DA , Shih J , et al. A gene signature predicting for survival in suboptimally debulked patients with ovarian cancer. Can Res. 2008;68:5478‐5486. 10.1158/0008-5472.CAN-07-6595 PMC7039050 18593951

[11] Cheon DJ , Tong Y , Sim MS , et al. A collagen‐remodeling gene signature regulated by TGF‐beta signaling is associated with metastasis and poor survival in serous ovarian cancer. Clin Cancer Res. 2014;20:711‐723. 24218511 10.1158/1078-0432.CCR-13-1256 PMC3946428

[12] Crijns AP , Fehrmann RS , de Jong S , et al. Survival‐related profile, pathways, and transcription factors in ovarian cancer. PLoS Med. 2009;6:e24. 19192944 10.1371/journal.pmed.1000024 PMC2634794

[13] Edgar R , Domrachev M , Lash AE . Gene expression omnibus: NCBI gene expression and hybridization array data repository. Nucleic Acids Res. 2002;30:207‐210. 11752295 10.1093/nar/30.1.207 PMC99122

[14] Hudson TJ , Anderson W , Artez A , et al. International network of cancer genome projects. Nature. 2010;464:993‐998. 20393554 10.1038/nature08987 PMC2902243

[15] Koti M , Gooding RJ , Nuin P , et al. Identification of the IGF1/PI3K/NF kappaB/ERK gene signalling networks associated with chemotherapy resistance and treatment response in high‐grade serous epithelial ovarian cancer. BMC Cancer. 2013;13:549. 24237932 10.1186/1471-2407-13-549 PMC3840597

[16] Yoshihara K , Tsunoda T , Shigemizu D , et al. High‐risk ovarian cancer based on 126‐gene expression signature is uniquely characterized by downregulation of antigen presentation pathway. Clin Cancer Res. 2012;18:1374‐1385. 22241791 10.1158/1078-0432.CCR-11-2725

[17] Uehara Y , Oda K , Ikeda Y , et al. Correction: integrated copy number and expression analysis identifies profiles of whole‐arm chromosomal alterations and subgroups with favorable outcome in ovarian clear cell carcinomas. PLoS ONE. 2015;10:e0132751. 26043110 10.1371/journal.pone.0128066 PMC4456367

[18] Mateescu B , Batista L , Cardon M , et al. miR‐141 and miR‐200a act on ovarian tumorigenesis by controlling oxidative stress response. Nat Med. 2011;17:1627‐1635. 22101765 10.1038/nm.2512

[19] Ritchie ME , Phipson B , Wu D , et al. limma powers differential expression analyses for RNA‐sequencing and microarray studies. Nucleic Acids Res. 2015;43:e47. 25605792 10.1093/nar/gkv007 PMC4402510

[20] Troyanskaya O , Cantor M , Sherlock G , et al. Missing value estimation methods for DNA microarrays. Bioinformatics (Oxford, England). 2001;17:520‐525. 10.1093/bioinformatics/17.6.520 11395428

[21] Chen C , Grennan K , Badner J , et al. Removing batch effects in analysis of expression microarray data: an evaluation of six batch adjustment methods. PLoS ONE. 2011;6:e17238. 21386892 10.1371/journal.pone.0017238 PMC3046121

[22] Huang da W , Sherman BT , Lempicki RA Systematic and integrative analysis of large gene lists using DAVID bioinformatics resources. Nat Protoc. 2009;4:44‐57. 19131956 10.1038/nprot.2008.211

[23] Kanehisa M , Furumichi M , Tanabe M , Sato Y , Morishima K . KEGG: new perspectives on genomes, pathways, diseases and drugs. Nucleic Acids Res. 2017;45:D353‐D361. 27899662 10.1093/nar/gkw1092 PMC5210567

[24] Moreno‐Betancur M , Sadaoui H , Piffaretti C , Rey G . Survival analysis with multiple causes of death: extending the competing risks model. Epidemiology (Cambridge, Mass.). 2017;28:12‐19. 10.1097/EDE.0000000000000531 27362647

[25] Zeng JH , Liang L , He RQ , et al. Comprehensive investigation of a novel differentially expressed lncRNA expression profile signature to assess the survival of patients with colorectal adenocarcinoma. Oncotarget. 2017;8:16811‐16828. 28187432 10.18632/oncotarget.15161 PMC5370003

[26] Heagerty PJ , Zheng Y . Survival model predictive accuracy and ROC curves. Biometrics. 2005;61:92‐105. 15737082 10.1111/j.0006-341X.2005.030814.x

[27] Leonard B , Starrett GJ , Maurer MJ , et al. APOBEC3G expression correlates with T‐cell infiltration and improved clinical outcomes in high‐grade serous ovarian carcinoma. Clin Cancer Res. 2016;22:4746‐4755. 27016308 10.1158/1078-0432.CCR-15-2910 PMC5026552

[28] Kobel M , Madore J , Ramus SJ , et al. Evidence for a time‐dependent association between FOLR1 expression and survival from ovarian carcinoma: implications for clinical testing. An Ovarian Tumour Tissue Analysis consortium study. Br J Cancer. 2014;111:2297‐2307. 25349970 10.1038/bjc.2014.567 PMC4264456

[29] Men CD , Liu QN , Ren Q . A prognostic 11 genes expression model for ovarian cancer. J Cell Biochem. 2018;119:1971‐1978. 28817186 10.1002/jcb.26358

[30] Malavasi F , Deaglio S , Damle R , Cutrona G , Ferrarini M , Chiorazzi N . CD38 and chronic lymphocytic leukemia: a decade later. Blood. 2011;118:3470‐3478. 21765022 10.1182/blood-2011-06-275610 PMC3574275

[31] Gao Y , Camacho LH , Mehta K . Retinoic acid‐induced CD38 antigen promotes leukemia cells attachment and interferon‐gamma/interleukin‐1beta‐dependent apoptosis of endothelial cells: implications in the etiology of retinoic acid syndrome. Leuk Res. 2007;31:455‐463. 16920192 10.1016/j.leukres.2006.07.004

[32] Colaianni G , Sun L , Di Benedetto A , et al. Bone marrow oxytocin mediates the anabolic action of estrogen on the skeleton. J Biol Chem. 2012;287:29159‐29167. 22761429 10.1074/jbc.M112.365049 PMC3436530

[33] Colaianni G , Di Benedetto A , Zhu LL , et al. Regulated production of the pituitary hormone oxytocin from murine and human osteoblasts. Biochem Biophys Res Comm. 2011;411:512‐515. 21741363 10.1016/j.bbrc.2011.06.158

[34] Hyndman DJ , Flynn TG . Sequence and expression levels in human tissues of a new member of the aldo‐keto reductase family. Biochem Biophys Acta. 1998;1399:198‐202. 9765596 10.1016/s0167-4781(98)00109-2

[35] Ohashi T , Idogawa M , Sasaki Y , Suzuki H , Tokino T . AKR1B10, a transcriptional target of p53, is downregulated in colorectal cancers associated with poor prognosis. Mol Cancer Res. 2013;11:1554‐1563. 24140838 10.1158/1541-7786.MCR-13-0330-T

[36] Yao HB , Xu Y , Chen LG , et al. AKR1B10, a good prognostic indicator in gastric cancer. Eur J Surg Oncol. 2014;40:318‐324. 24406159 10.1016/j.ejso.2013.12.014

[37] Ha SY , Song DH , Lee JJ , Lee HW , Cho SY , Park CK . High expression of aldo‐keto reductase 1B10 is an independent predictor of favorable prognosis in patients with hepatocellular carcinoma. Gut Liv. 2014;8:648‐654. 10.5009/gnl13406 PMC4215452 25287169

[38] Hong N , Kim BJ , Kim CH , et al. Low plasma level of leucine‐rich repeat‐containing 17 (LRRc17) is an independent and additive risk factor for osteoporotic fractures in postmenopausal women. J Bone Miner Res. 2016;31:2106‐2114. 27355564 10.1002/jbmr.2902

[39] Kim T , Kim K , Lee SH , et al. Identification of LRRc17 as a negative regulator of receptor activator of NF‐kappaB ligand (RANKL)‐induced osteoclast differentiation. J Biol Chem. 2009;284:15308‐15316. 19336404 10.1074/jbc.M807722200 PMC2685711

[40] Hsia LT , Ashley N , Ouaret D , Wang LM , Wilding J , Bodmer WF . Myofibroblasts are distinguished from activated skin fibroblasts by the expression of AOC3 and other associated markers. Proc Natl Acad Sci USA. 2016;113:E2162‐E2171. 27036009 10.1073/pnas.1603534113 PMC4839407

[41] Ochoa V , George AA , Nishi R , Whiteaker P . The prototoxin LYPD6B modulates heteromeric alpha3beta4‐containing nicotinic acetylcholine receptors, but not alpha7 homomers. FASEB J. 2016;30:1109‐1119. 26586467 10.1096/fj.15-274548 PMC4750422

[42] Chung BH , Mullegama S , Marshall CR , et al. Severe intellectual disability and autistic features associated with microduplication 2q23.1. Eur J Hum Genet. 2012;20:398‐403. 22085900 10.1038/ejhg.2011.199 PMC3306850

[43] Ishizuka T , Fujimori I , Kato M , et al. Human carboxymethylenebutenolidase as a bioactivating hydrolase of olmesartan medoxomil in liver and intestine. J Biol Chem. 2010;285:11892‐11902. 20177059 10.1074/jbc.M109.072629 PMC2852926

[44] Ishizuka T , Yoshigae Y , Murayama N , Izumi T . Different hydrolases involved in bioactivation of prodrug‐type angiotensin receptor blockers: carboxymethylenebutenolidase and carboxylesterase 1. Drug Metab Dispos. 2013;41:1888‐1895. 23946449 10.1124/dmd.113.053595

[45] Cantagrel V , Lossi AM , Boulanger S , et al. Disruption of a new X linked gene highly expressed in brain in a family with two mentally retarded males. J Med Genet. 2004;41:736‐742. 15466006 10.1136/jmg.2004.021626 PMC1735597

[46] de Lange IM , Helbig KL , Weckhuysen S , et al. novo mutations of KIAA2022 in females cause intellectual disability and intractable epilepsy. J Med Genet. 2016;53:850‐858. 27358180 10.1136/jmedgenet-2016-103909 PMC5264224

[47] Gilbert J , Man HY . The X‐linked autism protein KIAA2022/KIDLIA regulates neurite outgrowth via N‐cadherin and delta‐catenin signaling. eNeuro. 2016;3:1‐17. 10.1523/ENEURO.0238-16.2016 PMC5083950 27822498

[48] Kuroda Y , Ohashi I , Naruto T , et al. Delineation of the KIAA2022 mutation phenotype: two patients with X‐linked intellectual disability and distinctive features. Am J Med Genet A. 2015;167:1349‐1353. 25900396 10.1002/ajmg.a.37002

[49] Van Maldergem L , Hou Q , Kalscheuer VM , et al. Loss of function of KIAA2022 causes mild to severe intellectual disability with an autism spectrum disorder and impairs neurite outgrowth. Hum Mol Genet. 2013;22:3306‐3314. 23615299 10.1093/hmg/ddt187 PMC3723314

[50] Magome T , Hattori T , Taniguchi M , et al. XLMR protein related to neurite extension (Xpn/KIAA2022) regulates cell‐cell and cell‐matrix adhesion and migration. Neurochem Int. 2013;63:561‐569. 24071057 10.1016/j.neuint.2013.09.011

[51] Wang J , Liu D , Liang X , et al. Construction of a recombinant eukaryotic human ZHX1 gene expression plasmid and the role of ZHX1 in hepatocellular carcinoma. Mol Med Rep. 2013;8:1531‐1536. 24064680 10.3892/mmr.2013.1700

[52] Ma X , Huang M , Wang Z , Liu B , Zhu Z , Li C . ZHX1 inhibits gastric cancer cell growth through inducing cell‐cycle arrest and apoptosis. J Cancer. 2016;7:60‐68. 26722361 10.7150/jca.12973 PMC4679382

[53] Yue X , Zhang Z , Liang X , et al. Zinc fingers and homeoboxes 2 inhibits hepatocellular carcinoma cell proliferation and represses expression of Cyclins A and E. Gastroenterology. 2012;142:1559‐1570.e2. 22406477 10.1053/j.gastro.2012.02.049 PMC3367107

[54] Kwon RJ , Kim YH , Jeong DC , et al. Expression and prognostic significance of zinc fingers and homeoboxes family members in renal cell carcinoma. PLoS ONE. 2017;12:e0171036. 28152006 10.1371/journal.pone.0171036 PMC5289508

[55] Suehiro F , Nishimura M , Kawamoto T , et al. Impact of zinc fingers and homeoboxes 3 on the regulation of mesenchymal stem cell osteogenic differentiation. Stem Cells Dev. 2011;20:1539‐1547. 21174497 10.1089/scd.2010.0279

