# Supplementary material for: Bioinformatic profiling identifies a platinum‐resistant–related risk signature for ovarian cancer
Source: Cancer Med. 2019 Dec 19;9(3):1242–53. doi: 10.1002/cam4.2692 (PMC6997076; doi:10.1002/cam4.2692)
Supplement: Supplementary file 4 [file CAM4-9-1242-s004.doc]

| Category | Term | Count | % | PValue | Genes | List Total | Pop Hits | Pop Total | Fold Enrichment | Bonferroni | Benjamini | FDR |
| --- | --- | --- | --- | --- | --- | --- | --- | --- | --- | --- | --- | --- |
| KEGG_PATHWAY | hsa04062:Chemokine signaling pathway | 15 | 5.703422053 | 1.37E-06 | LYN, ADCY8, CXCL9, CXCL8, CCL8, CXCL11, STAT1, CCL5, CXCL10, CCL20, CXCL13, JAK2, GRK5, XCL1, AKT3 | 112 | 186 | 6910 | 4.975518433 | 2.72E-04 | 2.72E-04 | 0.001719187 |
| KEGG_PATHWAY | hsa04060:Cytokine-cytokine receptor interaction | 15 | 5.703422053 | 1.64E-05 | OSMR, CXCL9, CXCL8, CCL8, CCL5, IL7R, CXCL11, CXCL10, TNFSF10, TNFSF13B, CCL20, PRLR, CXCL13, CSF2RB, GHR | 112 | 230 | 6910 | 4.023680124 | 0.003248725 | 0.001625684 | 0.020562815 |
| KEGG_PATHWAY | hsa05164:Influenza A | 13 | 4.942965779 | 2.01E-05 | OAS3, RSAD2, CXCL8, OAS1, OAS2, STAT1, CCL5, CXCL10, DDX58, TNFSF10, HLA-DRB4, JAK2, AKT3 | 112 | 174 | 6910 | 4.609503284 | 0.003967269 | 0.001324176 | 0.025119323 |
| KEGG_PATHWAY | hsa04064:NF-kappa B signaling pathway | 8 | 3.041825095 | 4.63E-04 | DDX58, TNFSF13B, LYN, BCL2A1, CXCL8, LBP, BIRC3, TNFAIP3 | 112 | 87 | 6910 | 5.673234811 | 0.087669993 | 0.022677292 | 0.578191889 |
| KEGG_PATHWAY | hsa05168:Herpes simplex infection | 11 | 4.182509506 | 6.75E-04 | DDX58, SP100, TAP2, TAP1, OAS3, HLA-DRB4, OAS1, JAK2, OAS2, CCL5, STAT1 | 112 | 183 | 6910 | 3.708528493 | 0.125119124 | 0.026379332 | 0.84120293 |
| KEGG_PATHWAY | hsa05162:Measles | 9 | 3.422053232 | 0.001277005 | DDX58, TNFSF10, OAS3, OAS1, JAK2, OAS2, STAT1, TNFAIP3, AKT3 | 112 | 133 | 6910 | 4.174946294 | 0.223538718 | 0.041291376 | 1.586264089 |
| KEGG_PATHWAY | hsa04620:Toll-like receptor signaling pathway | 8 | 3.041825095 | 0.001505406 | CXCL9, CXCL8, LBP, CCL5, CXCL11, STAT1, AKT3, CXCL10 | 112 | 106 | 6910 | 4.656334232 | 0.257917612 | 0.041718375 | 1.867526117 |
| KEGG_PATHWAY | hsa04630:Jak-STAT signaling pathway | 9 | 3.422053232 | 0.002214259 | PRLR, OSMR, CSF2RB, JAK2, STAT1, IL7R, AKT3, GHR, IL13RA2 | 112 | 145 | 6910 | 3.829433498 | 0.355260796 | 0.053385811 | 2.7357307 |
| KEGG_PATHWAY | hsa05160:Hepatitis C | 8 | 3.041825095 | 0.005396677 | DDX58, OAS3, CXCL8, OAS1, OAS2, STAT1, PPP2R2C, AKT3 | 112 | 133 | 6910 | 3.711063373 | 0.657483578 | 0.112235175 | 6.54716381 |
| KEGG_PATHWAY | hsa04668:TNF signaling pathway | 7 | 2.661596958 | 0.006973741 | CCL20, MMP9, CCL5, BIRC3, TNFAIP3, AKT3, CXCL10 | 112 | 106 | 6910 | 4.074292453 | 0.749835812 | 0.12939228 | 8.384568186 |
| KEGG_PATHWAY | hsa04974:Protein digestion and absorption | 6 | 2.281368821 | 0.013318559 | COL9A3, SLC16A10, COL2A1, ATP1A2, COL4A6, COL4A5 | 112 | 88 | 6910 | 4.206574675 | 0.929686617 | 0.214429309 | 15.44596414 |
| KEGG_PATHWAY | hsa05340:Primary immunodeficiency | 4 | 1.520912548 | 0.016816954 | PTPRC, TAP2, TAP1, IL7R | 112 | 34 | 6910 | 7.258403361 | 0.965197744 | 0.244094887 | 19.12178898 |
| KEGG_PATHWAY | hsa00040:Pentose and glucuronate interconversions | 4 | 1.520912548 | 0.01960828 | CRYL1, UGT2B17, AKR1B10, AKR1B1 | 112 | 36 | 6910 | 6.85515873 | 0.980178902 | 0.260377995 | 21.94859116 |
| KEGG_PATHWAY | hsa04151:PI3K-Akt signaling pathway | 12 | 4.562737643 | 0.022216848 | LAMA1, PRLR, OSMR, FGF13, COL2A1, JAK2, IL7R, PPP2R2C, AKT3, COL4A6, GHR, COL4A5 | 112 | 345 | 6910 | 2.145962733 | 0.988304337 | 0.272217222 | 24.50785733 |
| KEGG_PATHWAY | hsa00982:Drug metabolism - cytochrome P450 | 5 | 1.901140684 | 0.023389027 | GSTM3, UGT2B17, MAOA, MAOB, ALDH3B2 | 112 | 68 | 6910 | 4.536502101 | 0.990776904 | 0.268313375 | 25.63254313 |
| KEGG_PATHWAY | hsa04514:Cell adhesion molecules (CAMs) | 7 | 2.661596958 | 0.026375867 | NCAM1, ALCAM, PTPRC, VTCN1, CD274, NLGN4X, HLA-DRB4 | 112 | 142 | 6910 | 3.041373239 | 0.994970979 | 0.281639055 | 28.42904323 |
| KEGG_PATHWAY | hsa05146:Amoebiasis | 6 | 2.281368821 | 0.027503746 | LAMA1, ARG2, CXCL8, COL2A1, COL4A6, COL4A5 | 112 | 106 | 6910 | 3.492250674 | 0.996002288 | 0.277346531 | 29.45963618 |
| KEGG_PATHWAY | hsa00360:Phenylalanine metabolism | 3 | 1.140684411 | 0.029721512 | MAOA, MAOB, ALDH3B2 | 112 | 17 | 6910 | 10.88760504 | 0.997456172 | 0.282436349 | 31.44639569 |
| KEGG_PATHWAY | hsa05145:Toxoplasmosis | 6 | 2.281368821 | 0.040899113 | LAMA1, HLA-DRB4, JAK2, BIRC3, STAT1, AKT3 | 112 | 118 | 6910 | 3.137106538 | 0.999743475 | 0.352847191 | 40.69902785 |
| KEGG_PATHWAY | hsa00330:Arginine and proline metabolism | 4 | 1.520912548 | 0.045849921 | ARG2, MAOA, MAOB, AGMAT | 112 | 50 | 6910 | 4.935714286 | 0.999907933 | 0.371644765 | 44.4176616 |
| KEGG_PATHWAY | hsa05222:Small cell lung cancer | 5 | 1.901140684 | 0.047379089 | LAMA1, BIRC3, AKT3, COL4A6, COL4A5 | 112 | 85 | 6910 | 3.629201681 | 0.999932983 | 0.367228725 | 45.52211072 |
| KEGG_PATHWAY | hsa05200:Pathways in cancer | 12 | 4.562737643 | 0.050131029 | LAMA1, ADCY8, MMP9, EGLN3, CXCL8, FGF13, BIRC3, STAT1, TCF7L1, AKT3, COL4A6, COL4A5 | 112 | 393 | 6910 | 1.883860414 | 0.999962207 | 0.370532504 | 47.45899216 |
| KEGG_PATHWAY | hsa04512:ECM-receptor interaction | 5 | 1.901140684 | 0.050851144 | LAMA1, CD44, COL2A1, COL4A6, COL4A5 | 112 | 87 | 6910 | 3.545771757 | 0.999967477 | 0.361915592 | 47.9552591 |
| KEGG_PATHWAY | hsa00340:Histidine metabolism | 3 | 1.140684411 | 0.051963963 | MAOA, MAOB, ALDH3B2 | 112 | 23 | 6910 | 8.047360248 | 0.999974218 | 0.356119935 | 48.71368157 |
| KEGG_PATHWAY | hsa05323:Rheumatoid arthritis | 5 | 1.901140684 | 0.05263841 | TNFSF13B, CCL20, HLA-DRB4, CXCL8, CCL5 | 112 | 88 | 6910 | 3.505478896 | 0.999977606 | 0.348364226 | 49.16837597 |
| KEGG_PATHWAY | hsa04621:NOD-like receptor signaling pathway | 4 | 1.520912548 | 0.05797991 | CXCL8, CCL5, BIRC3, TNFAIP3 | 112 | 55 | 6910 | 4.487012987 | 0.99999269 | 0.365461208 | 52.64060555 |
| KEGG_PATHWAY | hsa04066:HIF-1 signaling pathway | 5 | 1.901140684 | 0.072365358 | PDK1, CYBB, HKDC1, EGLN3, AKT3 | 112 | 98 | 6910 | 3.147776968 | 0.999999653 | 0.423546388 | 60.93597901 |
| KEGG_PATHWAY | hsa04210:Apoptosis | 4 | 1.520912548 | 0.077220418 | TNFSF10, CSF2RB, BIRC3, AKT3 | 112 | 62 | 6910 | 3.980414747 | 0.999999877 | 0.433509234 | 63.4186881 |
| KEGG_PATHWAY | hsa00052:Galactose metabolism | 3 | 1.140684411 | 0.083157184 | HKDC1, AKR1B10, AKR1B1 | 112 | 30 | 6910 | 6.169642857 | 0.999999966 | 0.447203753 | 66.25703303 |
| KEGG_PATHWAY | hsa05230:Central carbon metabolism in cancer | 4 | 1.520912548 | 0.083173509 | PDK1, HKDC1, PGAM2, AKT3 | 112 | 64 | 6910 | 3.856026786 | 0.999999966 | 0.43623876 | 66.26455027 |
| KEGG_PATHWAY | hsa04623:Cytosolic DNA-sensing pathway | 4 | 1.520912548 | 0.083173509 | DDX58, CCL5, AIM2, CXCL10 | 112 | 64 | 6910 | 3.856026786 | 0.999999966 | 0.43623876 | 66.26455027 |
| KEGG_PATHWAY | hsa04261:Adrenergic signaling in cardiomyocytes | 6 | 2.281368821 | 0.085364216 | ADCY8, PPP1R1A, SCN7A, ATP1A2, PPP2R2C, AKT3 | 112 | 146 | 6910 | 2.535469667 | 0.999999979 | 0.43442731 | 67.25947896 |
| KEGG_PATHWAY | hsa00051:Fructose and mannose metabolism | 3 | 1.140684411 | 0.092905268 | HKDC1, AKR1B10, AKR1B1 | 112 | 32 | 6910 | 5.784040179 | 0.999999996 | 0.453014592 | 70.481564 |
